# Porous and Ultra-Flexible Crosslinked MXene/Polyimide Composites for Multifunctional Electromagnetic Interference Shielding

**DOI:** 10.1007/s40820-022-00800-0

**Published:** 2022-02-09

**Authors:** Zhi-Hui Zeng, Na Wu, Jing-Jiang Wei, Yun-Fei Yang, Ting-Ting Wu, Bin Li, Stefanie Beatrice Hauser, Wei-Dong Yang, Jiu-Rong Liu, Shan-Yu Zhao

**Affiliations:** 1 Key Laboratory for Liquid-Solid Structural Evolution and Processing of Materials, Ministry of Education and School of Materials Science and Engineering, Shandong University, Jinan, 250061 People’s Republic of China; 2 Department of Chemistry and Applied Biosciences, ETH Zurich, CH-8093 Zurich, Switzerland; 3 Laboratory for Cellulose and Wood Materials, Swiss Federal Laboratories for Materials Science and Technology (Empa), 8600 Dübendorf, Switzerland; 4 Laboratory for Building Energy Materials and Components, Swiss Federal Laboratories for Materials Science and Technology (Empa), 8600 Dübendorf, Switzerland; 5 School of Aerospace Engineering and Applied Mechanics, Tongji University, Shanghai, 200092 People’s Republic of China

**Keywords:** MXene, Polyimide, Electromagnetic interference shielding, Heater, Sensor

## Abstract

Lightweight, ultra-flexible, and robust crosslinked transition metal carbide (Ti_3_C_2_ MXene) coated polyimide (PI) (C-MXene@PI) porous composites are manufactured via a scalable dip-coating followed by chemical crosslinking approach. In addition to the hydrophobicity, anti-oxidation and extreme-temperature stability, efficient utilization of the intrinsic conductivity of MXene, the interfacial polarization between MXene and PI, and the micrometer-sized pores of the composite foams are achieved. Consequently, the composites show a satisfactory X-band electromagnetic interference (EMI) shielding effectiveness of 22.5 to 62.5 dB at a density of 28.7 to 48.7 mg cm^−3^, leading to an excellent surface-specific SE of 21,317 dB cm^2^ g^−1^. Moreover, the composite foams exhibit excellent electrothermal performance as flexible heaters in terms of a prominent, rapid reproducible, and stable electrothermal effect at low voltages and superior heat performance and more uniform heat distribution compared with the commercial heaters composed of alloy plates. Furthermore, the composite foams are well attached on a human body to check their electromechanical sensing performance, demonstrating the sensitive and reliable detection of human motions as wearable sensors. The excellent EMI shielding performance and multifunctionalities, along with the facile and easy-to-scalable manufacturing techniques, imply promising perspectives of the porous C-MXene@PI composites in next-generation flexible electronics, aerospace, and smart devices.

**Supplementary Information:**

The online version contains supplementary material available at 10.1007/s40820-022-00800-0.

## Highlights

- Large-area, lightweight, ultra-flexible, and robust crosslinked MXene-coated PI porous composites were manufactured via a scalable and facile approach.
- In addition to the hydrophobicity, anti-oxidation and extreme-temperature stability, excellent electromagnetic interference shielding performance was achieved because of the high-efficiency utilization of the building units and microstructure.
- Moreover, the highly flexible composite foams exhibited excellent electrothermal and electromechanical sensing performance, demonstrating promising perspectives in next-generation flexible electronics, aerospace, and smart devices.

**Supplementary Information:**

The online version contains supplementary material available at 10.1007/s40820-022-00800-0.

## Graphical abstract

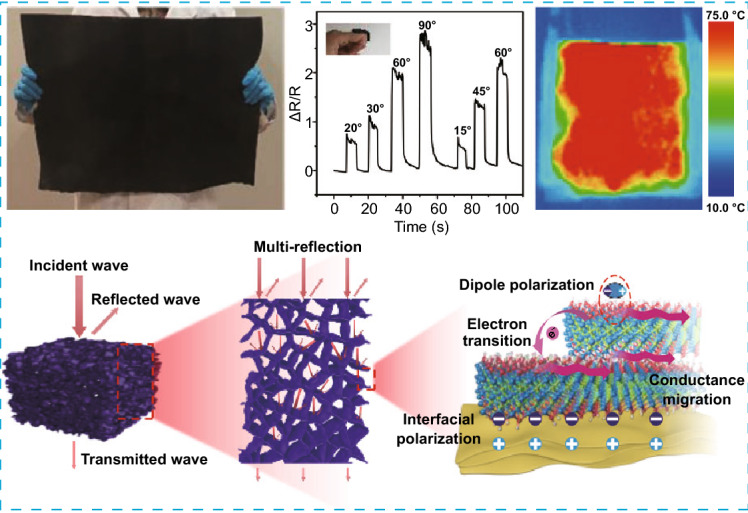

## Introduction

Advances in electromagnetic interference (EMI) shielding materials have sparked considerable attention in almost every electronics industry for attenuating electromagnetic radiation of complex electronic systems [1, 2]. High-performance EMI shields with lightweight, superior mechanical flexibility, and improved EMI shielding effectiveness (SE) are urgently required [3]. This promotes the development of numerous EMI shielding composites, involving porous architectures [4–7] or bulk shields [8–10], composed of lightweight, flexible polymers and highly conductive nanomaterials, such as carbon nanotubes (CNTs) [9, 11, 12], metal nanofibers [6, 13], and/or graphene layers [10, 14, 15]. Polymer matrices embedded with varieties of conductive nanofillers give rise to efficient conductive networks and abundant interfaces, which are beneficial for achieving high EMI shielding performance. Particularly, a porous architecture is remarkable in reducing the weight of the shields, and promoting multiple reflections (multi-reflections) of incident EM waves which consequently induces an increased EMI SE [4, 5, 11, 16–18]. For instance, we have reported a type of flexible CNT embedded polyurethane (PU) composite foams with EMI SE of 20–50 dB at density of merely 20–126 mg cm^−3^ [16]. After compressing the CNT/PU foams to exclude the micrometer-sized pores, the corresponding composites show significantly reduced EMI SE [18], confirming the significance of the porous structure in EMI shielding materials. Nevertheless, an efficient dispersion of the inherently inert conductive nanofillers in the polymer matrices accompanied by achieving effective porous structure to obtain high-performance EMI shielding composites in a facile, scalable preparation approach remains a great challenge [15, 19].

Transition metal carbides and/or nitrides (MXenes), a novel kind of two-dimensional (2D) nanomaterials, are famous for the “metal-like” conductivity, large specific surface, excellent mechanical properties, and easy processability in aqueous dispersion derived from the hydrophilic functional groups (–O, –OH, –F) [20, 21]. These provide huge potentials of MXene flakes for bottom-up construction of MXene-based EMI shielding macrostructures [22–24]. However, the interactions formed among MXene flakes are generally very weak, which is against the formation of robust pure MXene porous structures [20, 22, 25], and consequently hinder its practical applications. Polymers acting as efficient 'binder phase' are most widely employed to improve the mechanical strength and flexibility of MXene-based macrostructures [26, 27]. The poly (vinyl alcohol) (PVA) [28], sodium alginate (SA) [22], aramid fiber (ANF) [29, 30], and cellulose nanofiber (CNF) [26, 31–34] have been reported to achieve mechanical strong and flexible MXene-based composites. However, the introduced polymers are generally electrical insulator, which inevitably compromise the full utilization of the electrical conductivity and EMI shielding properties of MXenes. Poor temperature tolerance of commonly utilized polymers also restricts the wide range applications of the MXene-based EMI shielding composites [35]. Moreover, the high H_2_O/O_2_ permeability in polymer-MXene composites is critical for long-term durability regarding the poor oxidation stability of MXene [36–38]. In short, preparing lightweight, flexible, durable MXene-based porous architectures without compromising the excellent electrical conductivity of MXene in a scalable manufacturing approach remains challenging. In addition to EMI shielding performance, integrating multifunctionalities of devices is highly desirable with the rapid development of emerging multifunctional systems with internet of things (IoT) capabilities, such as wearable, flexible electronics including sensors [39–45] and heaters [46–52].

Here, we manufactured the lightweight, flexible, durable, and large-area crosslinked Ti_3_C_2_ MXene-coated polyimide (PI) (C-MXene@PI) composite foams based on a facile and scalable dip-coating followed by chemical crosslinking approach. Highly porous yet robust PI scaffolds rendered the composite foams with low density, ultra-flexibility, and extreme-temperatures tolerance. The entire covering of MXene flakes on continuous PI exoskeleton was beneficial for the retainment of high conductivity and EMI shielding performance of pristine MXene. Furthermore, the chemical crosslink of MXene (C-MXene) contributed to the hydrophobicity, waterproof capability, and the oxidation stability of the C-MXene@PI composite foams, promoting their high durability and reliability in practical applications. The 1.5-mm-thick C-MXene@PI composite foams with a density of 28.7 to 48.7 mg cm^−3^ also exhibited a satisfactory X-band EMI SE of 22.5 to 62.5 dB. Excellent specific SE (SSE, namely SE divided by the density) [4, 9, 19] and normalized surface-specific SE (SSE/*d*, namely SE divided by the density and thickness) [16, 22, 53] values up to 1,971 and 21,317 dB cm^2^ g^−1^ were also achieved, respectively, for the C-MXene@PI foams, which are significantly outperforming porous architectures including PI-based composites (Table 1) and other nanofiller embedded polymeric composites (Table S1). Combined with the theoretical calculation, we attributed the excellent EMI shielding performance to the synergistic coactions of micrometer-sized pores, the MXene-based conductive networks, and the interfacial polarization between the MXene and PI. Furthermore, we explored the excellent electrothermal and electromechanical sensing performance of the C-MXene@PI foams, showing the multifunctionalities and the potentials for multifunctional electronic devices. This work thus suggests a convenient, facile approach for large-scale manufacturing high-performance MXene-based porous composites with potential applications in EMI shielding of all kinds of complex electronic systems, and next-generation flexible electronic devices.

**Table 1:** EMI shielding performance of PI-based macrostructures and some typical MXene composites

Materials	EMI SE (dB)	Density (mg cm^−3^)	Thickness (mm)	SSE (dB cm^3^ g^−1^)	SSE/*d* (dB cm^2^ g^−1^)	Refs
C-MXene@PI foam	43.7	41.0	0.5	1066	21,317	This work
	80.8	41.0	3	1971	6569	
	62.52	48.7	1.5	1285	8567	
	60.04	43.0		1397	9315	
	59.17	41.0		1442	9612	
	52.51	38.0		1383	9217	
	45.54	35.6		1278	8519	
Ag NWs/PI foam	17–23.5	22	5	1068–772	2136 -1544	[59]
CNT/PI foam	41.1	32.1	2	1280	6402	[60]
MWCNT/PI	13.0–14.3	470	0.5	28–30	553–609	[69]
rGO/PI foam	13.7–15.1	460	0.5	30–33	596–657	[69]
MWCNT-CNT/rGO/PI foam	16.6–18.2	440	0.5	38–41	755–823	[69]
CNT/graphene/PI foam	28.2	20	2	1410	7050	[70]
Graphene/PI foam	22	280	0.8	78.6	982	[63]
Anisotropic graphene/PI foam	26.1–28.8	76	2.5	343–379	1373–1518	[71]
Graphene/PI foam	13.7 − 14.9	430	0.5	32–35	637 − 693	[72]
Graphene/PI film	31.3	~ 1200	0.151	26	1727	[73]
Carbon nanofiber/PI film	12	~ 1200	0.07	10	1429	[74]
Carbon nanofiber/carbon black/PI film	23.9	~ 1200	0.35	20	571	[75]
PI derived carbon foam	54	91	2	593	2965	[76]
Graphene/PI-derived carbon foam	24	720	0.024	33	13,888	[77]
MXene/PI porous film	54.5	390	0.09	140	15,527	[78]
MXene/nanocellulose film	24	2000	0.047	12	2647	[31]
MXene/CNF film	33	2477	0.0009	37	148,000	[57]
MXene/CNF foam	75	0.008	2	9320	46,600	[26]
MXene/PVA porous film	26	~ 545	0.1	48	4770	[28]
MXene/PVA foam	28	0.0108	5	2586	5136	[28]
MXene/ANF	28	1250	0.02	22	11,200	[29]
MXene/SA film	57	~ 2317	0.008	25	30,830	[22]

## Experimental

### Materials and Methods

#### Preparation of MXene Aqueous Dispersion

Aqueous dispersions of MXene were manufactured by etching and mechanical delamination process of the Ti_3_AlC_2_ MAX as shown in our previous work [26]. Briefly, 2.0 g Ti_3_AlC_2_ MAX (Laizhou Kai Kai Ceramic Materials Co., Ltd., China) was added to 40 mL hydrochloric acid (HCl, 9 M, Sigma-Aldrich, the USA) dissolved with 3.2 g lithium fluoride (LiF, Sigma-Aldrich, the USA). After the reaction at 35 ℃ for 24 h, the suspension was centrifuged at 3500 rpm and then redispersed to reach pH≈6. Afterward, the suspension was vigorously shaken for 30 min, and then, the supernatant MXene dispersion with a concentration of 0.1 wt% was obtained.

#### Preparation of C-MXene@PI Composite Foams

PI foams (Solimide® foam, BOYD Corporation GmbH) were immersed into the aforementioned MXene dispersion till the MXene dispersion was fully infiltrated into the PI foams and then dried in the 50 °C oven to get the MXene@PI composite foams. The dip-coating process was repeated, and the number was recorded. Afterward, the MXene@PI composite foams were chemically crosslinked by PMDI. Here, PMDI was first dissolved in acetonitrile/methyl caproate (4:1, v/v) solution at a volume ratio of 1:9, and then, the MXene@PI composite foams were immersed in this acetonitrile/methyl caproate (4:1, v/v) solution. After a reaction in the oven for 2 h at 70 °C followed by a acetone washing treatment, the freestanding C-MXene@PI composite foams were prepared.

### Characterization

Scanning electron microscopy (SEM, FEI NanoSEM 230), transmission electron microscopy (TEM, JEOL JEM2200fS) and atomic force microscopy (AFM, Bruker ICON3) were employed to characterize the morphology and microstructure. A drop shape analyzer (DSA 30, Krüss, Germany) was utilized to measure the water contact angles (CA). A FTIR spectrometer (PerkinElmer Spectrum Two) with an attenuated total reflection accessory was used to perform the FTIR measurements. The resistances (*R*) were measured in a four-probe method by a Keithley 4200 electrometer so as to calculate the electrical conductivity (*δ*). EMI SE in the frequency range of 8.2–12.4 GHz (X-band) was measured by a vector network analyzer (Agilent 8517A) in the waveguide method. More than three specimens were tested for each component. The S-parameters were recorded and used to calculate the SE_T_, SE_R_, and SE_A_. To evaluate the electrothermal performance, various DC voltages were applied to the 10L MXene@PI composite foams using a DC-regulated power supply. The temperature of the sample was measured by a digital thermometer (UT325) with its T-type thermocouple contacting the surface of the sample. The electromechanical response of the composite foams was obtained by measuring the resistance change using the Keithley 4200-SCS electrometer in a two-probe method.

## Results and Discussion

### Preparation and Structure of the Composite Foam

**Fig. 1:**
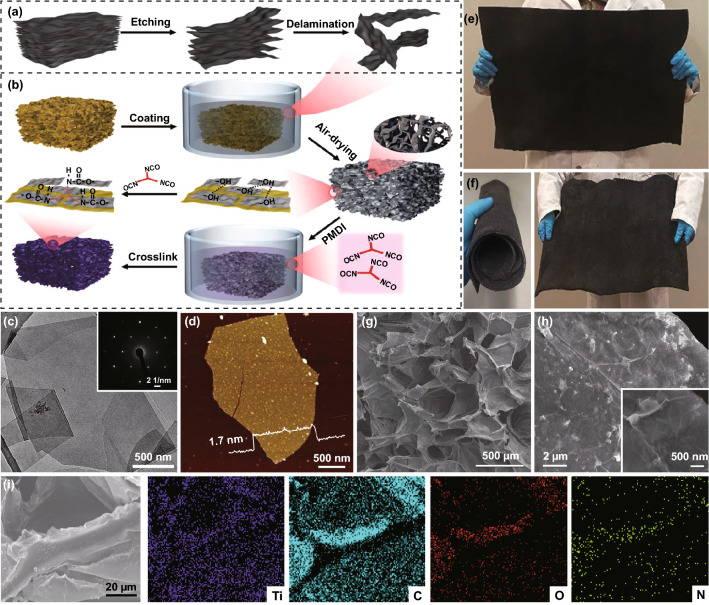
Scalable manufacturing of C-MXene@PI composite foams: schematic of preparation process of **a** MXene flakes and **b** C-MXene@PI composite foams, **c** TEM (inset shows electron diffraction image) and **d** AFM (inset shows the height profile of a monolayer) images of the MXene flakes. **e, f** Photographs of freestanding and large-area (~ 60 × 60 cm^2^) C-MXene@PI composite foams before and after rolling, showing the mechanical robustness and flexibility. SEM images of the **g** micrometer-sized pores and **h** cell walls, and **i** Ti-, C-, O-, and N- element mappings of the C-MXene@PI composite foams

The manufacturing process of C-MXene@PI composite foams is schematically displayed (Fig. 1a, b). First, a stable Ti_3_C_2_T_x_ MXene (T represents the surface hydrophilic functional groups (-OH, -O, and -F)) in an aqueous dispersion was prepared by etching and delamination of the precursor Ti_3_AlC_2_ MAX with a compact rocklike microstructure (Figs. 1a and S1a) [26, 27]. After etching of Al layers of MAX precursor [26, 54], multilayer Ti_3_C_2_T_x_ (m-Ti_3_C_2_T_x_) was obtained (Fig. S1b). Subsequent repeated washing with deionized water and vigorous shaking to swell the m-Ti_3_C_2_T_x_ were carried out, leading to the preparation of aqueous dispersion composed of delaminated MXene nanoflakes. A high Zeta potential of around − 40 mV showed stability of the MXene in aqueous dispersion (Fig. S1c). Dominant single-layer MXene flakes with an average lateral size of around 2 μm and a hexagonal atomic structure were observed in the TEM and electron diffraction images, respectively (Fig. 1c). AFM image is further provided, and the thickness of ~ 1.7 nm can be identified for a single MXene flake (Fig. 1d); this is consistent with the previous reports [26, 27, 55]. The pure PI foams showed micrometer-sized pores and smooth pore cell surfaces (Fig. S2a), which interconnected and sustained the low-density (~ 25 mg cm^−3^) porous scaffolds. The strong hydrogen bonding interactions formed between the imide rings of PI and MXene nanoflakes allowed for MXene adhered well to PI skeletons, leading to a successful preparation of MXene-coated PI (MXene@PI) composite foams. The coated MXene flakes interconnected to form substantial conductive networks, which were instrumental in significantly improving the electrical conductivity of the PI scaffolds [56]. The MXene nanoflakes were further crosslinked by the chemical crosslinking agent poly ((phenyl isocyanate)-co-formaldehyde) (PMDI). The isocyanate group of PMDI efficiently reacts with the hydroxyl groups on MXene, leading to the formation of strong peptide bonds. A typical, large-area (60 × 60 cm^2^) crosslinked MXene-coated PI (C-MXene@PI) composite foams exhibiting excellent mechanical robustness and flexibility were manufactured (Fig. 1e, f). Compared with the pure PI foams, the C-MXene@PI foams showed the same micrometer-sized pores yet rougher cell walls due to the crosslinked MXene layers on the PI cell walls (Figs. 1g, h and S2b). Element mappings of the C-MXene@PI composite foams further demonstrated the successful composite and that the pore walls were coated with numerous MXene flakes (Fig. 1i).

**Fig. 2:**
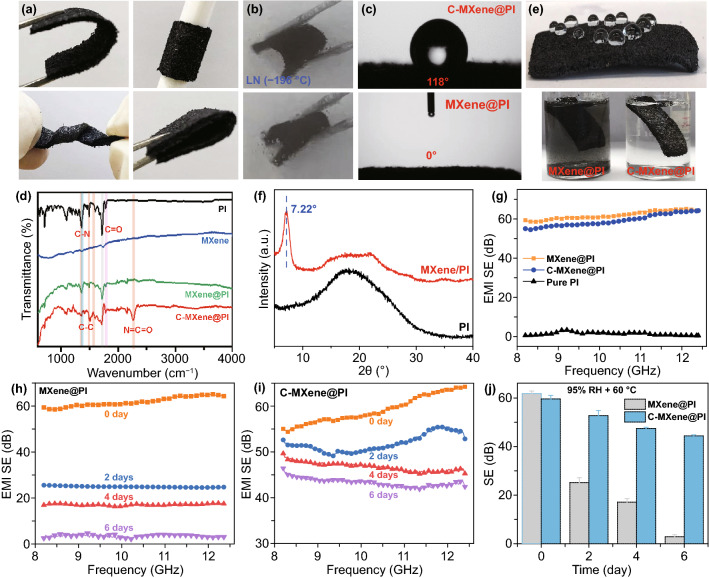
Photographs of **a** the C-MXene@PI composite foams showing ultra-flexible performance including bendability, rollability, twistability, foldability at room temperature and **b** the composite soaked in liquid nitrogen (LN, -196 °C) with maintained mechanical flexibility. **c** Contact angles of the MXene@PI and C-MXene@PI composite foams. **d** FTIR curves of PI, MXene@PI, and C-MXene@PI foams. **e** Photograph of C-MXene@PI foams with several drops of water on the surface, as well as the MXene@PI and C-MXene@PI foams after ultrasonic treatment in water for 30 min, showing that the C-MXene@PI foams are waterproof. **f** XRD patterns of the PI and C-MXene@PI foams. **g** X-band EMI SE of the PI, MXene@PI, and C-MXene@PI foams. X-band EMI SE of the **h** MXene@PI and **i** C-MXene@PI composite foams after stored in a 95% RH environment and a temperature of 60 °C for different days and **j** the corresponding change of SE as a function of time. In the first day (d = 0), the foams were in a dry state

The interfacial interactions between MXene and PI as well as the flexible and robust PI scaffolds endowed the C-MXene@PI composite foams with excellent mechanical flexibility including bendability, rollability, twistability, and even foldability (Fig. 2a). Even though soaked in the liquid nitrogen at an extreme temperature of -196 ℃, the ultra-flexibility of the C-MXene@PI composite foam was still maintained (Fig. 2b, Video S1). In contrast, the commercial PU foams upon bending broke easily in this low-temperature condition (Fig. S3, Video S2), proving the significance of PI scaffolds on our flexible and reliable composite foams especially for extreme conditions. Furthermore, the chemical crosslinking of MXene flakes increased the water contact angle from 0° to 118° (Figs. 2c and S4a, Video S3), which was mainly ascribed to the introduction of the hydrophobic backbone in the PMDI [57]. As displayed in FTIR curves (Figs. 2d and S5), the characteristic peaks of polyimide (1720, 1780 cm^−1^ for C=O and 1380 cm^−1^ for C-N) presented in both pure polyimide and MXene-coated polyimide foams (Fig. 2d). After incubated with chemical crosslinker PMDI, the emblematic bands (around 1410 and 1504 cm^−1^) of benzene ring in PMDI can be observed on the spectra of C-MXene@PI (Fig. S5a). The new appearance of characteristic benzene ring vibrations and a CO–NH mode (1700 cm^−1^ for C=O in the urethane bonding, Fig. S5b) in C-MXene@PI showed the successful chemical coating of PMDI on the MXene. Consequently, such hydrophobic coating led to excellent stability and water resistance of the MXenes composites. After ultrasonic treatment for 20 min of the MXene@PI and C-MXene@PI composite foams were immersed in water, the former deteriorated completely, while the latter stayed as one piece, and there was no MXene detachment (Figs. 2e and S4b).

### EMI Shielding Performance of the Composite Foam

In addition to the significant color change of the PI scaffolds upon the coating of MXene nanoflakes (Fig. S6), the 2-theta angle of ~ 7.22° in XRD pattern corresponding to the interlayer gaps of 1.2 nm between the MXene nanoflakes further shows a well-preserved structure of MXene flakes (Fig. 2f), which indicates a high utilization efficiency of MXenes’ conductivity and EMI shielding properties [22]. The coated MXene flakes on PI skeleton led to a remarkable increase in X-band EMI SE from 1.4 to 60 dB for the MXene@PI composite foams at a thickness of 1.5 mm. It is worth noting that EMI SE describes the attenuation capability of samples to the incident EM waves (Table S2), and an EMI SE value of 20 dB corresponding to a 99% attenuation of the waves is generally required for commercial applications [13, 19, 22]. Apart from the satisfactory EMI SE, our composite foams showed larger SE values than other PI-based composites (Table 1) and typical conductive nanomaterial embedded porous composites ever reported at similar thicknesses [58], *e.g.*, 5-mm-thick AgNW/PI [59], 2-mm-thick CNT/PI [60], 2.3-mm-thick CNT/PU [16], 2.3-mm-thick graphene/PEI [61] composite foams reached SE values up to 12.8, 41.1, 50.5, and 12.8 dB, respectively. Chemical crosslinking of MXene flakes shown ignorable influence on the electrical conductivity and EMI SE properties (Figs. 2g and S7a), demonstrating the potentials of our C-MXene@PI composite foams as high-performance EMI shielding materials. More intriguingly, the chemical crosslink efficiently improved the oxidation stability of the C-MXene@PI composites in H_2_O/O_2_ environment (Fig. 2h–i), which is crucial for long durability of the composites in practical applications. After being stored in a 95% RH environment and a temperature of 60 ℃ for 2 days, EMI SE of the MXene@PI composite foams remarkably decreased and the shielding effect almost disappeared after 6 days in the same condition. In contrast, the C-MXene@PI composite foams maintained a high EMI SE of 44.4 dB after being stored in the same condition for 6 days (Fig. 2j). The behavior for resistance change of the composite foam stored in such condition is consistent with that of EMI SE (Fig. S7b). Therefore, stable, durable C-MXene@PI composite foams with a remarkable EMI SE were achieved successfully.

**Fig. 3:**
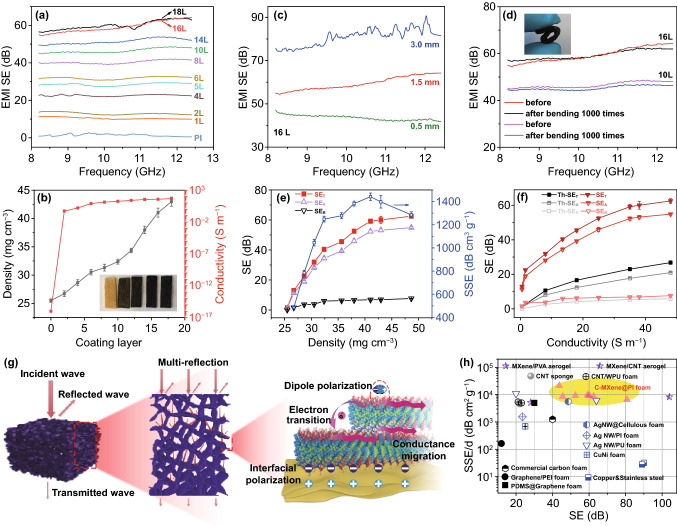
**a** X-band EMI SE of C-MXene@PI composite foams with various dip-coating layers. **b** Density and electrical conductivity of the C-MXene@PI composite foams as a function of the dip-coating layers. **c** The 16L C-MXene@PI composite foams at various thicknesses, and **d** the 10L and 16L C-MXene@PI composite foams before and after 1000 times bending treatment, showing the good shielding stability against mechanical deformations. **e** EMI shielding performance (SE_T_, SE_A_, and SE_R_) and SSE of C-MXene@PI composite foams as function of sample density. **f** Theoretically calculated EMI shielding performance (Th-SE_T_, Th-SE_A_, and Th-SE_R_) of a bulk based on the electrical conductivity of corresponding C-MXene@PI foams and the comparison to the experimentally tested EMI shielding performance (SE_T_, SE_A_, and SE_R_) of the C-MXene@PI composite foams. **g** Schematic showing the proposed EMI shielding mechanism of the C-MXene@PI composite foams for ultrahigh EMI shielding performance. **h** EMI shielding performance comparison of the C-MXene@PI composite foams with other typical porous architectures: SSE/*d* values of typical EMI shielding macrostructures with corresponding SE values (the experimental data and source reference used in this figure are summarized in Table S1)

In the facile and scalable “layer-by-layer” dip-coating approach, EMI SE of C-MXene@PI composite foams can be widely controlled by adjusting the coating layers/times with MXene suspensions (Fig. 3a). The EMI SE of the 1.5-mm-thick porous composites increased with increasing coating layers, *e.g.*, EMI SE reached a commercial SE value after a 4 times coating (namely 4 Layers MXene, 4L), while it increased to 41 and 60 dB after 10 and 16 times coatings, respectively. Nevertheless, when the composites were coated by 18 (18L) and more times, the increase of EMI SE reached a plateau. To better realize the behavior, we concluded the density and electrical conductivity of the C-MXene@PI composite foams as a function of coating layer (Fig. 3b). The density increased with the increasing of the coating layers, which correlates to the increased MXene loading and related thickness on PI scaffolds. According to the MXene-PDMI layer thickness (around 0.8 μm) identified from the SEM image of a 14L C-MXene/PI composite foam (Fig. S2b), we can easily calculate the MXene-PDMI thickness on the PI skeletons based on the measured density of the composite foams. Furthermore, a clear observation in Fig. 3a, 1L C-MXene@PI composite foams can already form sufficient conductive paths due to the efficient adhesion of MXene on the interconnected PI cell walls. This led to the rapid transformation from insulation to conduction of the porous scaffolds. With increasing of coating layers, more MXene nanoflakes were stacked on the cell walls, forming improved conductive networks, which eventually affected the electrical conductivity and EMI SE of the composites. However, the changes of electrical conductivity, as well as EMI SE, were not remarkably, especially above 10L coatings, which indicates good compatibility between MXene and PI fibers, and abundant intact conductive paths have been well established during the initial several coating rounds. Additionally, the EMI SE was controlled by adjusting the thickness of the C-MXene@PI composite foams, *e.g.*, 16L composites reached an EMI SE value of 43.7 to 80.8 dB at a thickness of 0.5 to 3.0 mm (Fig. 3c). Moreover, the porous composites showed excellent resistance and EMI SE stability upon mechanical deformation, the resistance and EMI SE remained almost constant after the sample was bent for 1000 cycles (Figs. 3d and S7c). Briefly, the controllable and stable EMI shielding performance further demonstrates the great promises of our C-MXene@PI foams for practical applications.

In order to realize the EMI shielding mechanism of the C-MXene@PI composite foams, we measured the shielding by absorption (SE_A_) and reflection (SE_R_) (Fig. 3e). Generally, EMI shielding performance of conductive porous composites is influenced by the reflection, absorption, and multi-reflections, corresponding to the mobile charge carriers, electric dipoles, and interior interfaces/surfaces, respectively [19, 56, 62]. The micrometer-sized pores induced more reflections or scatterings of incident EM waves, which had more interactions with the pore walls in the C-MXene@PI composite foams, efficiently increasing SE_A_ [16–18, 63]. The large mismatch of conductivity in the interfaces between the MXene and PI also led to high interfacial polarization [8], which combined with the abundant charge carriers from MXenes [23, 63, 64], resulting in increased SE_A_ of the pore walls. In addition, the MXene terminal functional groups were considered to give rise to electric dipoles under the electric field of the EM wave [17, 22, 26], improving the SE_A_ of the MXene-based composites. Thus, the SE_A_ dominated the total SE (SE_T_), which sum up both SE_R_ and SE_A_. With increasing density of the C-MXene@PI composite foams derived from increased MXene loadings, SE_T_ and SE_A_ of the composites thus increased significantly and achieved maximum values of 62.5 and 54.9 dB, respectively, at a density of 48.7 mg cm^−3^. Furthermore, we had theoretically calculated the EMI shielding performance (Th-SE_T_, Th-SE_A_, and Th-SE_R_) (the details of the theoretical calculation method are shown in our previous work [13, 16]) of a homogenous shield based on the conductivities derived from our C-MXene@PI composite foams (Fig. 3f). Obviously, apart from the similar SE_R_ values, Th-SE_A_ was obviously lower than the experimentally tested SE_A_. This was attributed to that the introduced multi-reflections caused by the porous structure gave rise to more interactions of the incident waves with the MXene/PI composite cell walls, which effectively absorbed the EM waves derived from the conduction and polarization loss capability. As a consequent, the experimentally tested SE_T_ was much higher than the Th-SE_T_. In short, we could efficiently demonstrate that the high EMI shielding performance of our C-MXene@PI composite foams is attributed to the synergistic interactions among MXene, PI skeleton and the porous structure (Fig. 3g).

To better realize the lightweight EMI shielding architectures, we calculated the SSE of the C-MXene@PI composite foams with various densities (Fig. 3e). Interestingly, the SSE initially had a significant increase with increasing density, and it reached the extremum of 1,442 dB cm^3^ g^−1^ at a density of 41 mg cm^−3^, and a further increase in density led to the drop of SSE. This illustrated that suitable coating times of the MXene nanoflakes on the PI skeleton were vital for better utilizing the MXene for the EMI shielding porous architectures. Notably, the EMI SE reached a value of 59.2 dB at such a large SSE for the C-MXene@PI composite foams, which showed opposite behavior with other composites having a higher SSE value with decreasing density or SE values [5, 16, 18]. Here, the efficient design of our C-MXene@PI composite foams contributed both higher SE and SSE values, which significantly outperformed that of other typical nanofiller embedded polymeric porous composites at similar thickness (Table 1), *e.g.*, EMI SE was around 22, 41, and 23 dB for the graphene/PI [65], CNT/PI [58], and CNT/PU [16] porous composites at SSE values of 78.6, 1280, and 1184 dB cm^3^ g^−1^, respectively. Certainly, a higher SE associated with a minimum material consumption is crucial for achieving high-performance EMI shielding architectures, and thus, SSE/*d* was proposed in our previous work [13, 16, 53] to efficiently evaluate the lightweight EMI shields. Herein, the SSE/*d* of C-MXene@PI composite foams could reach 9,612 and 21,317 dB cm^2^ g^−1^ at a SE of 59.2 and 43.7 dB, respectively. This performance was superior to that of other porous EMI materials including commercial carbon foams, carbon nanotube sponges, graphene-based foams, metal-based foams, and other MXene-based porous composites (Fig. 3h, Table S1). Particularly, the EMI shielding performance among the PI-based architectures is compared in Table 1, effectively proving the superiority of EMI performance of C-MXene@PI composite foams. Combined with the service stability, as well as the energy-efficient, facile, and scalable preparation method, the lightweight, ultra-flexible, and robust C-MXene@PI composite foams with outstanding EMI shielding performance show huge potentials in the applications of aerospace, portable electronics and smart wearable devices.

### Electrothermal Performance of the Composite Foam

**Fig. 4:**
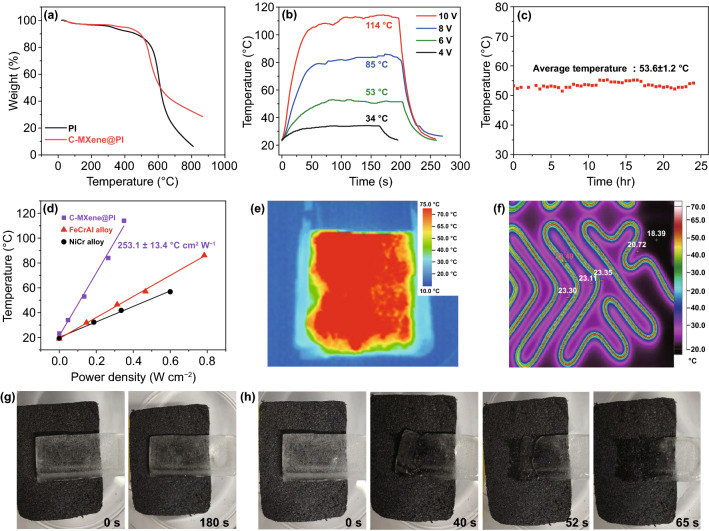
Electrothermal performance of the C-MXene@PI composite foams. **a** TGA curves of the C-MXene@PI composite and pure PI foams in air, **b** the electrothermal curves of the C-MXene@PI composite foams (4 × 4 × 0.15 cm^3^) at various voltages, and **c** life-time tests of the C-MXene@PI composite foams for 24 h. **d** The steady-state temperature versus input power density curves of the C-MXene@PI composite foams and commercial Fe–Cr–Al and Ni–Cr alloy plates. Thermal image map of the **e** C-MXene@PI composite foams and **f** commercial metal-based heaters, showing the uniform temperature of the C-MXene@PI composite foams. Electrothermal deicing performance. Demonstration for a deicing application of the C-MXene@PI composites: **g** the composite without applied voltage and **h** the composite at an applied voltage of 8 V

Besides the EMI application, multifunctionality is always expected for such lightweight conductors. First extension was on electrothermal materials, which convert electric power to thermal energy have attracted more and more attention due to the rapid development of electronic engineering [47, 48, 51, 52]. Traditional electrothermal materials, including Fe–Cr–Al or Ni–Cr-based alloys and electrothermal ceramics, suffer from complicated manufacturing process, heavy weight, inflexible shape and low heating efficiency. Here, thermogravimetric analysis (TGA) curves of pure PI and C-MXene@PI foams showed the good thermal stability of the PI-based architectures (Fig. 4a), in contrast to most polymers. Combined with the efficient conductive networks, the ultra-flexible C-MXene@PI composite foams showed great potentials for lightweight and stable electrothermal heaters. Therefore, various DC voltages were applied to the C-MXene@PI composite foams (4 × 4 × 0.15 cm^3^), and the currents flowing through the foams were observed. Figure 4b showed the effect of these low DC voltages on the temperature of the 10L C-MXene@PI composite foams. The composites had evident, stable, and reversible electrothermal effect at these voltages. For example, the composite foams with a large size of 40 × 40 × 1.5 mm^3^ were able to reach up to 34, 53, 84, and 114 °C in tens of seconds at 4, 6, 8, and 10 V, respectively. At the same time, the life-time tests for the composite heaters had been carried out. As shown in Fig. 4c, a DC voltage of 6 V was applied to the composite heaters for 24 h, and the temperature was measured every half hour. It was found that the steady-state heating of the samples, which further demonstrated a stable electrothermal effect of the composite. A stable and remarkable electrothermal performance of the C-MXene@PI composite foams was achieved at low DC voltages, demonstrating the possibility and prospects in various application areas.

The equilibrium temperature of the composites showed a linear relation with the input power densities (Fig. 4d). The slope of the steady-state temperature versus input power density revealed the heat performance (*H*_p_ = d*T*/d*P*) of the composite heater [53, 56], as the greater the *H*_p_ value, the higher the steady-state temperature of the heater, at certain initial temperature and input electric power. The *H*_p_ value of the C-MXene@PI composite samples was 253.1 °C cm^2^ W^−1^, higher than that of other systems including CNTs, rGOs and metal thin films, as shown in Table 2. In addition, the composite foam heaters reached higher temperature than the typical commercial metal electrothermal heater Fe–Cr–Al and Ni–Cr alloy plates at the same input power density (Fig. 4e, f). In other words, the input power required for the composite foams was less than that of the commercial alloy-based heaters in order to reach the same temperature with the same heater area. More intriguingly, arising from the uniform distribution of MXene on PI skeleton, the lightweight C-MXene@PI composite foam heaters had an even temperature distribution, which could not be achieved by the heavy commercial heaters composed of metal alloy strips which easily had local overheating (Fig. 4e, f). Based on the high electrothermal performance, a demonstration for a high-performance deicing or anti-icing application at low voltages is well shown (Fig. 3g, h). Combining the excellent flexibility, high heat performance, low working voltage and uniform temperature distribution under 12 V in the range of automobile power supply, and the robust behavior demonstrated by the life-time test, the C-MXene@PI composite foam heaters show extended prospects in practical applications and can be produced on large scale for health-care-related products. The combination of the excellent MI shielding and electrothermal performance further efficiently demonstrates the great application potentials of the C-MXene@PI composites as multifunctional devices.

**Table 2:** Comparison of *H*_p_ of typical heaters

Heating material	Substrate	*H*_p_ [°C cm^2^ W^−1^]	Refs.	Notes
SWCNTs	Glass	212, 200	[51, 52]	Spray method
	/	137	[67]	Dip-coating method
MWCNTs	PET	94	[68]	Yarn from vertical-aligned
rGO	Glass	163	[66]	Spin-coated GO/heat-treated
Ag	/	92	[52]	Silver paste
		54		Silver paste
Pt	none	65		Sputtering
Fe–Cr-Al alloy	none	85	This work	Commercial production
Ni–Cr alloy	none	62		
MXene	PI foam	253	This study	Scalable dip-coating approach

### Electromechanical Sensing Performance of the Composite Foam

**Fig. 5:**
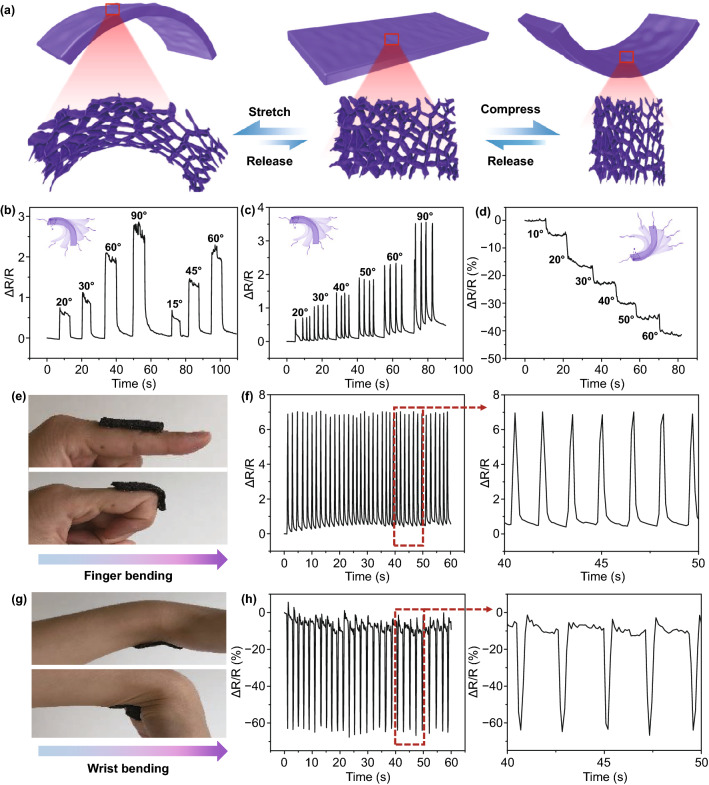
Detections of human motions for the flexible C-MXene@PI composite foams as electromechanical strain sensors. **a** Bending-induced stretch and compression modes of the C-MXene@PI composite foams. **b, c** The resistance changes (ΔR/R = (R1-R)/R, where R1 and R are the resistance and initial resistance of the sample, resistance) of the C-MXene@PI composite foam upon various bending angle induced stretch. **d** The resistance changes of the C-MXene@PI composite foam upon various bending angle induced compress. **e, f** Detections of finger bending activities with cyclic bending which induces resistance change of the C-MXene@PI composite foam. **g, h** Detections of wrist bending activities with cyclic bending which induces resistance change of the C-MXene@PI composite foam

Since our C-MXene@PI composite foams are highly flexible and robust, as the second extension, they can be effortlessly attached to human body as wearable sensors for detecting the human motions [43–45]. As the three-dimensional (3D) composite samples were bent, stretching and compressing were caused in different sides (Fig. 5a). The stretched and compressed side of the 3D composite sensors corresponds to the formation of less and more conductive paths in the C-MXene@PI composites, respectively, which correspond to the increased and decreased electrical resistance, respectively (Fig. 5b–d). The gauge factor corresponding to the sensitivity of the foam sensors is also calculated based on the bending angle (Fig. S8), and we can conclude that the bending-induced stretching leads to a high sensitivity. When the composites bent to an angel and then kept steady, the resistance of the sensor initially changed due to the deformation and then kept stable. Moreover, a larger relative resistance change is observed for a larger bending angle, showing the usefulness of our sensors in the detection of various human motions, from small strain to large strain activities. Additionally, even the bending speed of the sample was very fast, the change of the resistance could be well detected and distinguished. Therefore, the detected relatively resistance change’s signal of the sensor attached on a finger bending circularly can be displayed. When the finger bent to a fixed angel, then returned periodically, the resistance of the C-MXene@PI composite foam sensor increased and recovered accordingly and periodically (Fig. 5e, f), ascribing to the stretching caused by the bend of the composite foams. In contrast, when the composite foam was attached on the wrist bending circularly, the resistance decreased and recovered periodically due to the compression caused by the bend of the composite foams (Fig. 5g, h). According to the measured electrical signals from the composite sensor, we can easily deduce that in the testing period, the finger or wrist repeatedly bends and moves back quickly with similar amplitude, and number and of bends in each interval.

## Conclusion

Lightweight, ultra-flexible, and large-area C-MXene@PI composite foams were prepared in a facile and scalable dip-coating followed by a chemical crosslinking approach. The coactions of the highly porous yet robust PI scaffolds, coated MXene nanoflakes, and the effective chemical crosslink treatment rendered the C-MXene@PI foams with hydrophobicity, waterproof capability, anti-oxidation and extreme-temperature stability. The combination also benefits high utilization of the MXene electrical conductivity, and an interfacial polarization between the MXene and PI. As-prepared C-MXene@PI foams show an ultrahigh EMI shielding performance accompanied by excellent durability and reliability. The 1.5-mm-thick C-MXene@PI composite foams exhibit a satisfactory X-band EMI SE of 22.5 to 62.5 dB at a density of 28.7 to 48.7 mg cm^−3^. Excellent SSE and SSE/*d* values of 1,971 dB cm^3^ g^−1^ and 21,317 dB cm^2^ g^−1^ are achieved, respectively, for the C-MXene@PI foams, significantly surpassing other typical porous architectures. Furthermore, two possible extensions of the C-MXene@PI foams were exanimated. A rapid reproducible, and stable electrothermal effect of C-MXene@PI foams at low voltages is demonstrated, which shows ultrahigh heat performance and uniform heat distribution. Secondly, the excellent performance of the C-MXene@PI foams as flexible wearable sensors is well demonstrated with sensitive and reliable detection capability of human motions. The integration of ultrahigh EMI shielding, electrothermal, and electromechanical sensing performances of the C-MXene@PI foams as well as their facile and scalable production approach suggest the promising application potentials for next-generation flexible and multifunctional electronic devices and aerospace.

## Supplementary Information

Below is the link to the electronic supplementary material.

Supplementary file1 (PDF 738 kb)

Supplementary file2 (MP4 26392 kb)

Supplementary file3 (MP4 16249 kb)

Supplementary file4 (MP4 9097 kb)
