# Supplementary material for: Porous and Ultra-Flexible Crosslinked MXene/Polyimide Composites for Multifunctional Electromagnetic Interference Shielding
Source: Nanomicro Lett. 2022 Feb 9;14:59. doi: 10.1007/s40820-022-00800-0 (PMC8828842; doi:10.1007/s40820-022-00800-0)
Supplement: Supplementary file 1 — Supplementary file1 (PDF 738 kb) [file 40820_2022_800_MOESM1_ESM.pdf]

Supporting Information for

Porous and Ultra-Flexible Crosslinked MXene/Polyimide Composites for Multifunctional Electromagnetic Interference Shielding

Zhi-Hui Zeng[1, #, *], Na Wu[2, #], Jing-Jiang Wei[3], Yun-Fei Yang[1], Ting-Ting Wu[4], Bin Li[1], Stefanie Beatrice Hauser[4], Wei-Dong Yang[5], Jiu-Rong Liu[1, *], Shan-Yu Zhao[4, *]

[1]School of Materials Science and Engineering, Shandong University, Jinan 250061, P. R. China

[2]Department of Chemistry and Applied Biosciences, ETH Zurich, CH-8093 Zurich, Switzerland

[3]Laboratory for Cellulose and Wood Materials, Swiss Federal Laboratories for Materials Science and Technology (Empa), 8600 Dübendorf, Switzerland

[4]Laboratory for Building Energy Materials and Components, Swiss Federal Laboratories for Materials Science and Technology (Empa), 8600 Dübendorf, Switzerland

[5]School of Aerospace Engineering and Applied Mechanics, Tongji University, Shanghai 200092, P. R. China

[#]Zhi-Hui Zeng and Na Wu contributed equally to this work

*Corresponding authors. E-mail: zhihui.zeng@sdu.edu.cn (Z.-H Zeng), jrliu@sdu.edu.cn (J.-R Liu), shanyu.zhao@empa.ch (S.-Y Zhao)

Supplementary Figures and Tables

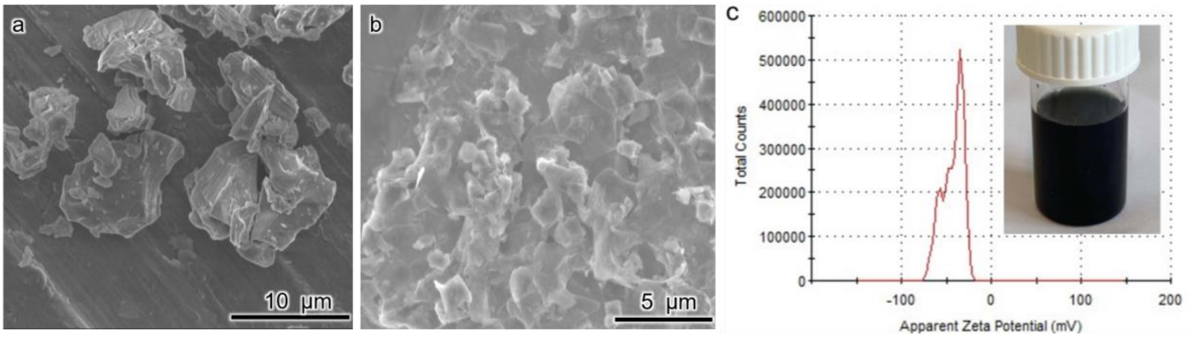

Fig. S1 SEM images of the (**a**) MAX precursor and (**b**) m-$Ti_3C_2T_x$. More details can be seen from our previous work [S26]. (**c**) Zeta potential of as-prepared MXene and photograph of the MXene aqueous dispersion, showing the stability of the dispersion

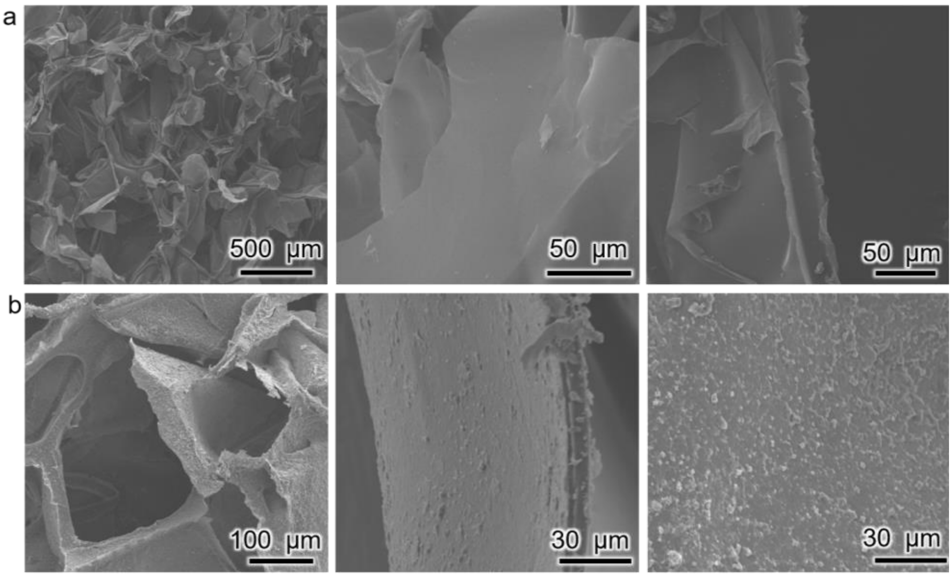

Fig. S2 SEM images of (**a**) pure PI foams and (**b**) C-MXene@PI composite (coated 14 times with MXene) foams

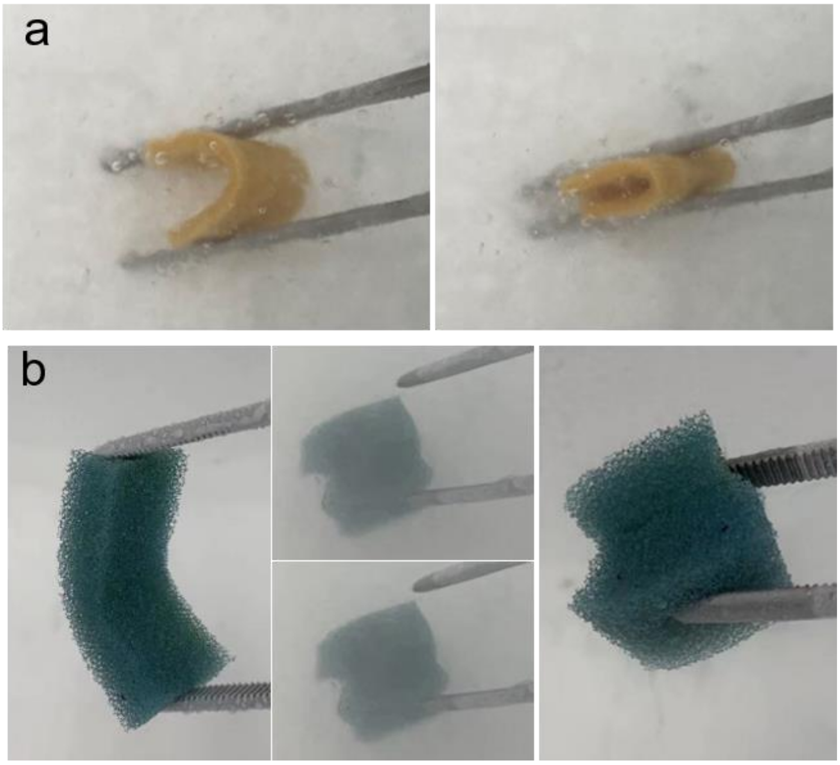

Fig. S3 Photographs of the (**a**) as-prepared pure PI foams and (**b**) commercial PU foams soaked in the liquid nitrogen, showing the robustness and flexibility of the PI foams while the PU foams broke easily after bent in liquid nitrogen

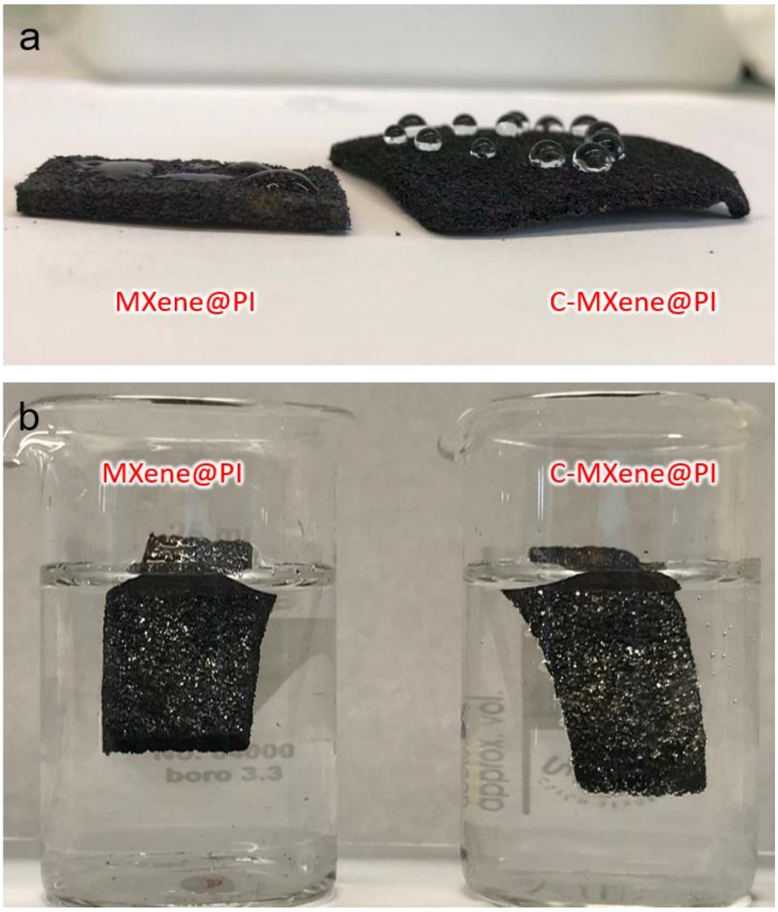

Fig. S4 (**a**) Photographs of the MXene@PI and C-MXene@PI composite foams showing the surface wettability with water. (**b**) Photographs of the MXene@PI and C-MXene@PI composite foams when immersed in water without further treatment

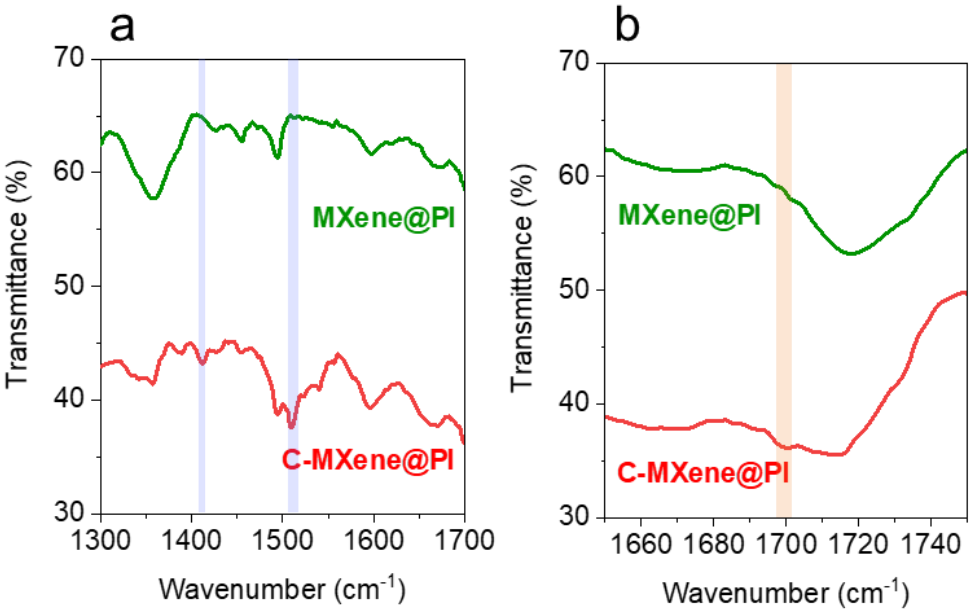

Fig. S5 (**a**) Emblematic benzene bands on PMDI. (**b**) The carbonyl stretching region of an urethane mode

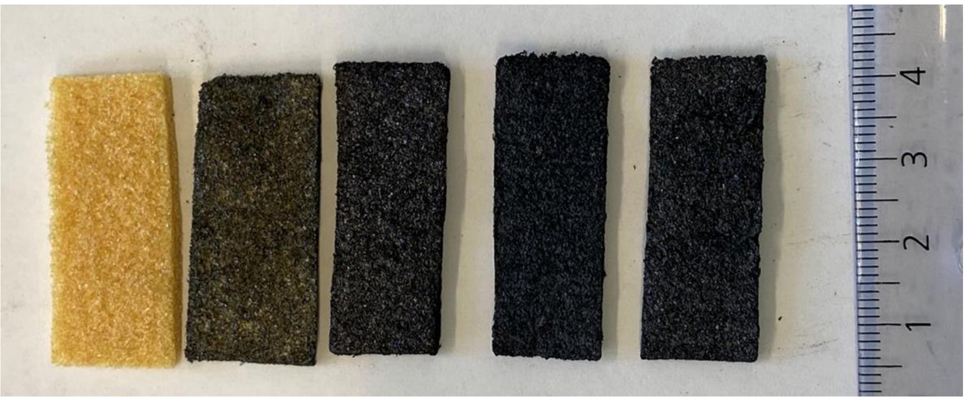

Fig. S6 Photograph of the C-MXene@PI composite foams with various coating times/layers (2L, 4L, 10L, 16L C-MXene@PI foams are shown from left to right)

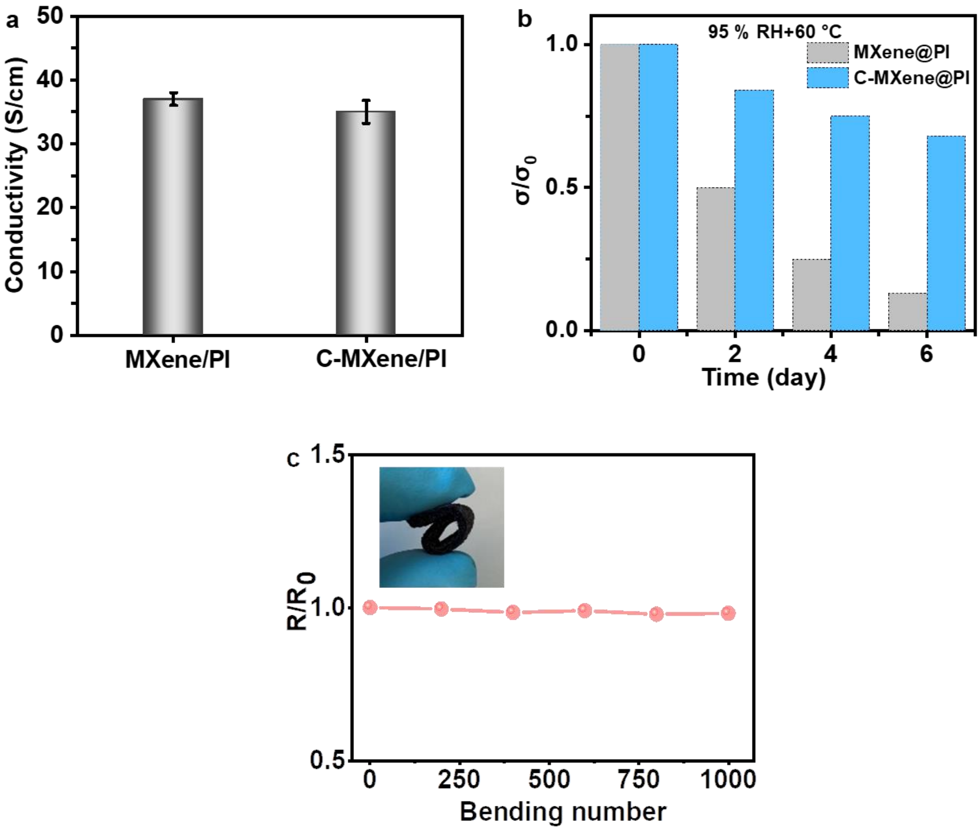

Fig. S7 (**a**) Electrical conductivity of the 14L MXene/PI composites before and after crosslinking treatment. (**b**) Conductivity (σ) change of MXene/PI and C-MXene/PI composite foams after stored in a 95% RH environment and a temperature of 60 °C for different days. In the first day (d = 0), the foams were in a dry state. (**c**) Resistance change of 16L C-MXene/PI composites upon the cyclic bending treatment

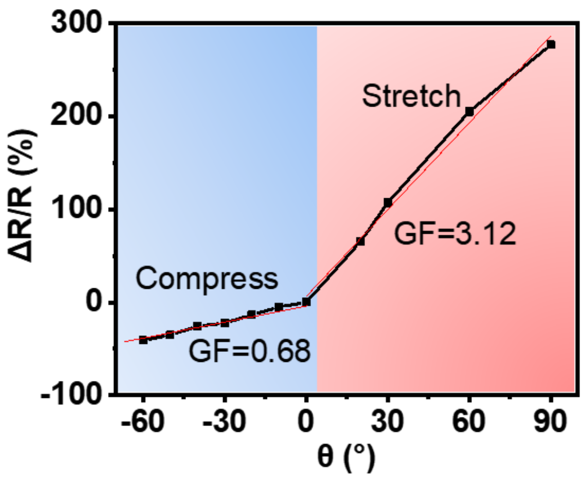

Fig. S8 Bending angle (θ)-resistance change ((ΔR/R) curve shows the sensitivity of the PI foam-based sensors upon bending induced compression and stretching. (Gauge factor is the slope of the curves)

Table S1 EMI shielding performance of typically porous shielding materials

Materials	EMI SE (dB)	Density (mg/cm^3)	Thickness (mm)	SSE (dB·cm^3/g)	SSE/*d* (dB·cm^2/g)	Refs.
**MXene-based porous shields**						
**C-MXene@PI foam**	**43.7**	**41.0**	**0.5**	**1066**	**21317**	**This work**
	**80.8**	**41.0**	**3**	**1971**	**6569**	
	**62.52**	**48.7**		**1285**	**8567**	
	**60.04**	**43.0**	**1.5**	**1397**	**9315**	
	**59.17**	**41.0**		**1442**	**9612**	
	**52.51**	**38.0**		**1383**	**9217**	
	**45.54**	**35.6**		**1278**	**8519**	
Porous MXene film	32	390	0.006	82	137000	[S1]
MXene/CNT aerogel	104	42	3	2476	8254	[S2]
MXene-POSS-NH_2 aerogel	34.5	/	2	/	/	[S3]
MXene/PVA foam	28	10.8	5	2586	5136	[S4]
**Carbon-based porous shields**						
CNT/PI foam	41.1	32.1	2	1280.4	6402	[S5]
CNF/PS foam	19		/	/	/	[S6]
CNT/PS foam	19	574	/	33.1	/	[S7]
Graphene/PVDF foam	28		/	/	/	[S8]
Graphene/PMMA foam	19	792	2.4	24	100	[S9]
Graphene/PS foam	29	450	2.5	64.4	258	[S10]
Graphene /PEI foam	9-12.8	~290	2.3	31– 44	135–192	[S11]
Graphene@Fe_3O_4/PEI foam	15-18	400	2.5	37.5–44	150–176	[S12]
CF/PP foam	25	735	3.1	34	109	[S13]

Stainless-steel fiber/PP foam	48	640	3.1	75	242	[S14]
MWCNT/PLA foam	23	299	2.5	77	308	[S15]
MWCNT/PVDF foam	57	750	2	76	380	[S16]
MWCNT/WPU foam	23.0	20	2.3	1148	4991	[S17]
	21.1	39	1	541	5410	
	50.5	120	2.3	401	1743	
MWCNT/cellulose aerogel	20-35	~37-47	2.5	425-944	1700-3776	[S18]
Cellulose aerogel coated with MWCNT	35-40	~69-75	2.5	466-519	1864-2078	
Graphene/PI foam	22	280	0.8	78.6	982	[S19]
Graphene foam based PDMS foam	30	60	1	~500	~5000	[S20]
Graphene foam/CNT/PDMS	75	90	2	833	4165	[S21]
Graphene-coated PU foam	19.9	30.0	20	663.3	3320	[S22]
Graphene foam coated with PEDOT:PSS	69.1	22.1	1.5	3124	20837	[S23]
Graphene based composite aerogel	37	70	3	529	1762	[S24]
Sponged-supported RGO aerogel	24	16.7	12	1437	1198	[S25]
CNT/multi-layered graphene foam	~38	5.8	1.6	6600	~40000	[S26]
Graphene/cellulose-derived carbon foam	47.8	2.8	5.0	16890	33780	[S27]
Graphene/lignin-derived carbon aerogels	23.2	2.5	2	9280	46400	[S28]
	14.3	2.5	1	5720	57200	
Graphene aerogel	22.3	4.5	2	4956	24778	
Carbon foam-CNT/carbon fiber foam	21	12.4	5.0	1690	3370	[S29]
CNT mat	30	/	0.001	/	/	[S30]
CF mat	23	/	0.06	/	/	[S31]
Ni/CF mat	29	/	0.06	/	/	
Fe_3O_4/CNF mat	68	/	0.7	/	/	[S32]
CNF mat	81.1	219	4.6	370	804.3	[S33]
	52.2	134	2.9	390	1361.6	
Graphene/CNA	58.4		2.0	/	/	[S34]
Carbon/Graphene foam	24	721	0.024	33.3	13889	[S35]
Graphene foam	25.2	60	0.3	420.0	14000	[S36]
Phthalonitrile-based carbon foam	51.2	150	2	341.1	1707	[S37]

Commercial carbon foam	40	166	2	241	1250	[S38]
CNT sponge	22	20	2.38	1100	4622	[S39]
**Metal-based porous shields**						
CuNi foam	15-25	~240	1.5	63–104	420–690	[S40]
CuNi-CNT foam	40-54.6	~230	1.5	174–237	116–1580	
Porous cellulose papers coated with Ag NWs	48.6	530	0.164	91.7	5584	[S41]
Ag NWs/PI foam	17-23.5	22	5	1068-772	2136 -1544	[S42]
Ag NWs/WPU foam	20.0-64.0	8.0	2.3	2500-1422	10970-6184	[S43]
Ag NW@C hybrid sponge	37.9	3.8	1	9921	99214	[S44]
Cu NWs aerogels	~17		9.46	/	/	
Cu NW@ graphene aerogels	52.5	166	9.46	3170	3921.8	[S45]

Table S2 The corresponding power attenuation and transmission efficiency (T) of EM waves for shields with various EMI SE values

EMI SE (dB)	Attenuation (%)	T (%)
10	90	10
20	99	1
30	99.9	0.1
40	99.99	0.01
50	99.999	0.001
60	99.9999	0.0001
70	99.99999	0.00001
80	99.999999	0.000001

Supplementary References

[S1] J. Liu, H.B. Zhang, R. Sun, Y. Liu, Z. Liu et al., Hydrophobic, flexible, and lightweight MXene foams for high-performance electromagnetic-interference shielding. Adv. Mater. **29**(38), 1702367 (2017). https://doi.org/10.1002/adma.201702367

[S2] R. Bian, G. He, W. Zhi, S. Xiang, T. Wang et al., Ultralight MXene-based aerogels with high electromagnetic interference shielding performance. J. Mater. Chem. C **7**(3), 474-478 (**2019**). https://doi.org/10.1039/C8TC04795B

[S3] P. Sambyal, A. Iqbal, J. Hong, H. Kim, M.K. Kim et al., Ultralight and mechanically robust $Ti_3C_2T_x$ hybrid aerogel reinforced by carbon nanotubes for electromagnetic interference shielding. ACS Appl. Mater. Interfaces **11**(41), 38046-38054 (2019). https://doi.org/10.1021/acsami.9b12550

[S4] S. Shi, B. Qian, X. Wu, H. Sun, H. Wang et al., Self assembly of MXene-surfactants at liquid-liquid interfaces: from structured liquids to 3D aerogels. Angew. Chem. Int. Ed. **58**(50), 18171-18176 (2019). https://doi.org/10.1002/anie.201908402

[S5] H. Xu, X. Yin, X. Li, M. Li, S. Liang et al., Lightweight Ti_2CT_x MXene/poly(vinyl alcohol) composite foams for electromagnetic wave shielding with absorption-dominated feature. ACS Appl. Mater. Interface **11**(10), 10198-10207 (2019). https://doi.org/10.1021/acsami.8b21671

[S6] Y.Y. Wang, Z.H. Zhou, C.G. Zhou, W.J. Sun, J.F. Gao et al., Lightweight and robust carbon nanotube/polyimide foam for efficient and heat-resistant electromagnetic interference shielding and microwave absorption. ACS Appl. Mater. Interface **12**(7), 8704-8712 (2020). https://doi.org/10.1021/acsami.9b21048

[S7] Y. Yang, M.C. Gupta, K.L. Dudley, R.W. Lawrence, Conductive carbon nanofiber–polymer foam structures. Adv. Mater. **17**(16), 1999-2003 (2005). https://doi.org/10.1002/adma.200500615

[S8] Y. Yang, M.C. Gupta, K.L. Dudley, R.W. Lawrence, Novel carbon nanotube−polystyrene foam composites for electromagnetic interference shielding. Nano Lett. **5**(11), 2131-2134 (2005). https://doi.org/10.1021/nl051375r

[S9] V. Eswaraiah, V. Sankaranarayanan, S. Ramaprabhu, Functionalized graphene-PVDF foam composites for EMI shielding. Macromol. Mater. Eng. **296**(10), 894-898 (2011). https://doi.org/10.1002/mame.201100035

[S10] H.B. Zhang, Q. Yan, W.G. Zheng, Z. He, Z.Z. Yu, Tough graphene-polymer microcellular foams for electromagnetic interference shielding. ACS Appl. Mater. Interfaces **3**(3), 918-924 (2011). https://doi.org/10.1021/am200021v

[S11] D.X. Yan, P.G. Ren, H. Pang, Q. Fu, M.B. Yang et al., Efficient electromagnetic interference shielding of lightweight graphene/polystyrene composite. J. Mater. Chem. **22**(36), 18772-18774 (2012). https://doi.org/10.1039/C2JM32692B

[S12] J. Ling, W. Zhai, W. Feng, B. Shen, J. Zhang et al., Facile preparation of lightweight microcellular polyetherimide/graphene composite foams for electromagnetic interference shielding. ACS Appl. Mater. Interfaces **5**(7), 2677-2684 (2013). https://doi.org/10.1021/am303289m

[S13] B. Shen, W. Zhai, M. Tao, J. Ling, W. Zheng, Lightweight, multifunctional polyetherimide/graphene@Fe_3O_4 composite foams for shielding of electromagnetic pollution. ACS Appl. Mater. Interfaces **5**(21), 11383-11391 (2013). https://doi.org/10.1021/am4036527

[S14] A. Ameli, P.U. Jung, C.B. Park, Electrical properties and electromagnetic interference shielding effectiveness of polypropylene/carbon fiber composite foams. Carbon **60**, 379-391(2013). https://doi.org/10.1016/j.carbon.2013.04.050

[S15] A. Ameli, M. Nofar, S. Wang, C.B. Park, Lightweight polypropylene/stainless-steel fiber composite foams with low percolation for efficient electromagnetic interference shielding. ACS Appl. Mater. Interfaces **6**(14), 11091-11100 (2014). https://doi.org/10.1021/am500445g

[S16] T. Kuang, L. Chang, F. Chen, Y. Sheng, D. Fu et al., Facile preparation of lightweight high-strength biodegradable polymer/multi-walled carbon nanotubes nanocomposite foams for electromagnetic interference shielding. Carbon **105**, 305-313 (2016). https://doi.org/10.1016/j.carbon.2016.04.052

[S17] H. Wang, K. Zheng, X. Zhang, X. Ding, Z. Zhang et al., 3D network porous polymeric composites with outstanding electromagnetic interference shielding. Compos. Sci. Technol. **125**, 22-29 (2016). https://doi.org/10.1016/j.compscitech.2016.01.007

[S18] Z. Zeng, H. Jin, M. Chen, W. Li, L. Zhou et al., Lightweight and anisotropic porous MWCNT/WPU composites for ultrahigh performance electromagnetic interference shielding. Adv. Funct. Mater. **26**(2), 303-310 (2016). https://doi.org/10.1002/adfm.201503579

[S19] L.Q. Zhang, S.G. Yang, L. Li, B. Yang, H.D. Huang et al., Ultralight cellulose porous composites with manipulated porous structure and carbon nanotube distribution for promising electromagnetic interference shielding. ACS Appl. Mater. Interface **10**(46), 40156-40167 (2018). https://doi.org/10.1021/acsami.8b14738

[S20] Y. Li, X. Pei, B. Shen, W. Zhai, L. Zhang et al., Polyimide/graphene composite foam sheets with ultrahigh thermostability for electromagnetic interference shielding. RSC Adv. **5**(31), 24342-14351 (2015). https://doi.org/10.1039/C4RA16421K

[S21] Z. Chen, C. Xu, C. Ma, W. Ren, H.M. Cheng, Ligthweight and flexible graphene foam composites for high-performance electromagnetic interference shielding. Adv. Mater. **25**(9), 1296-1300 (2013). https://doi.org/10.1002/adma.201204196

[S22] X. Sun, X. Liu, X. Shen, Y. Wu, Z. Wang et al., Graphene foam/carbon nanotube/poly(dimethyl siloxane) composites for exceptional microwave shielding. Comp. Part A Appl. Sci. Manuf. **85**, 199-206 (2016). https://doi.org/10.1016/j.compositesa.2016.03.009

[S23] B. Shen, Y. Li, W. Zhai, W. Zheng, Compressible graphene-coated polymer foams with ultralow density for adjustable electromagnetic interference (EMI) shielding. ACS Appl. Mater. Interfaces **8**(12), 8050-8057 (2016). https://doi.org/10.1021/acsami.5b11715

[S24] Y. Wu, Z. Wang, X. Liu, X. Shen, Q. Zheng et al., Ultralight graphene foam/conductive polymer composites for exceptional electromagnetic interference shielding. ACS Appl. Mater. Interfaces **9**(10), 9059-9069 (2017). https://doi.org/10.1021/acsami.7b01017

[S25] W.L. Song, X.T. Guan, L.Z. Fan, W.Q. Cao, C.Y. Wang et al., Tuning three-dimensional textures with graphene aerogels for ultra-light flexible graphene/texture composites of effective electromagnetic shielding. Carbon **93**, 151-160 (2015). https://doi.org/10.1016/j.carbon.2015.05.033

[S26] C. Liu, S. Ye, J. Feng, The preparation of compressible and fire-resistant sponge-supported reduced graphene oxide aerogel for electromagnetic interference shielding. Chem. Asian J. **11**(18), 2586-2593 (2016). https://doi.org/10.1002/asia.201600905

[S27] Q. Song, F. Ye, X. Yin, W. Li, H. Li et al., Carbon nanotube-multilayered graphene edge plane core-shell hybrid foams for ultrahigh-performance electromagnetic-

interference shielding. Adv. Mater. **29**(31), 1701583 (2017). https://doi.org/10.1002/adma.201701583

[S28] Y.J. Wan, P.L. Zhu, S.H. Yu, R. Sun, C.P. Wong et al., Ultralight, super-elastic and volume-preserving cellulose fiber/graphene aerogel for high-performance electromagnetic interference shielding. Carbon **115**, 629-639 (2017). https://doi.org/10.1016/j.carbon.2017.01.054

[S29] Z. Zeng, C. Wang, Y. Zhang, P. Wang, S.I.S. Shahabadi et al., Ultralight and highly elastic graphene/lignin-derived carbon nanocomposite aerogels with ultrahigh electromagnetic interference shielding performance. ACS Appl. Mater. Interfaces **10**(9), 8205-8213 (2018). https://doi.org/10.1021/acsami.7b19427

[S30] O. Pitkanen, J. Tolvanen, I. Szenti, A. Kukovecz, J. Hannu et al., Lightweight hierarchical carbon nanocomposites with highly efficient and tunable electromagnetic interference shielding properties. ACS Appl. Mater. Interfaces **11**(21), 19331-19338 (2019). https://doi.org/10.1021/acsami.9b02309

[S31] Z.P. Wu, T. Liu, D.M. Chen, G. Wu, Q. Wang et al., A facile method to improving the electromagnetic interference shielding of a free-standing and foldable carbon nanotube mat. RSC Adv. **6**(67), 62485–62490 (2016). https://doi.org/10.1039/c6ra11507a

[S32] T. Kim, D.D.L. Chung, Mats and fabrics for electromagnetic interference shielding. J. Mater. Eng. Perform. **15**, 295-298 (2016). https://doi.org/10.1361/105994906X108594

[S33] M. Bayat, H. Yang, F. Ko, D. Michelson, A. Mei, Electromagnetic interference shielding effectiveness of hybrid multifunctional Fe_3O_4/carbon nanofiber composite. Polymer **55**(3), 936-943 (2014). https://doi.org/10.1016/j.polymer.2013.12.042

[S34] X. Hong, D.D.L. Chung, Carbon nanofiber mats for electromagnetic interference shielding. Carbon **111**, 529-537 (2017). https://doi.org/10.1016/j.carbon.2016.10.031

[S35] C. Wan, J. Li, Graphene oxide/cellulose aerogels nanocomposite: preparation, pyrolysis, and application for electromagnetic interference shielding. Carbhydr. Polym. **150**, 172-179 (2016). https://doi.org/10.1016/j.carbpol.2016.05.051

[S36] Y. Li, B. Shen, X. Pei, Y. Zhang, D. Yi et al., Ultrathin carbon foams for effective electromagnetic interference shielding. Carbon **100**, 375-385 (2016). https://doi.org/10.1016/j.carbon.2016.01.030

[S37] B. Shen, Y. Li, D. Yi, W. Zhai, X. Wei et al., Microcellular graphene foam for improved broadband electromagnetic interference shielding. Carbon **102**, 154-160 (2016). https://doi.org/10.1016/j.carbon.2016.02.040

[S38] L. Zhang, M. Liu, S. Roy, E. Chu, K. See et al., Phthalonitrile-based carbon foam with high specific mechanical strength and superior electromagnetic interference shielding performance. ACS Appl. Mater. Interfaces **8**(11), 7422-7430 (2016). https://doi.org/10.1021/acsami.5b12072

[S39] F. Moglie, D. Micheli, S. Laurenzi, M. Marchetti, V.M. Primiani, Electromagnetic shielding performance of carbon foams. Carbon **50**, 1972-1980 (2012). https://doi.org/10.1016/j.carbon.2011.12.053

[S40] M. Crespo, M. González, A. L. Elías, L.P. Rajukumar, J. Baselga et al., Ultra-light carbon nanotube sponge as an efficient electromagnetic shielding material in the GHz range. Phys. Stat. Sol. RRL **8**(8), 698-704 (2014). https://doi.org/10.1002/pssr.201409151

[S41] K. Ji, H. Zhao, J. Zhang, J. Chen, Z. Dai, Fabrication and electromagnetic interference shielding performance of open-cell foam of a Cu–Ni alloy integrated with CNTs. Appl. Surf. Sci. **311**, 351-356 (2014). https://doi.org/10.1016/j.apsusc.2014.05.067

[S42] T.W. Lee, S.E. Lee, Y.G. Jeong, Highly effective electromagnetic interference shielding materials based on silver nanowire/cellulose papers. ACS Appl. Mater. Interfaces **8**(20), 13123-13132 (2016). https://doi.org/10.1021/acsami.6b02218

[S43] J. Ma, M. Zhan, K. Wang, Ultralightweight silver nanowires hybrid polyimide composite foams for high-performance electromagnetic interference shielding. ACS Appl. Mater. Interfaces **7**(1), 563-576 (2015). https://doi.org/10.1021/am5067095

[S44] Z. Zeng, M. Chen, Y. Pei, S.I.S. Shahabadi, B. Che et al., Ultra-light and flexible polyurethane/silver nanowire nanocomposites with unidirectional pores for highly effective electromagnetic shielding. ACS Appl. Mater. Interfaces **9**(37), 32211-32219 (2017). https://doi.org/10.1021/acsami.7b07643

[S45] Y.J. Wan, P.L. Zhu, S.H. Yu, R. Sun, C.P. Wong et al., Anticorrosive, ultralight, and flexible carbon-wrapped metallic nanowire hybrid sponges for highly efficient electromagnetic interference shielding. Small **14**(27), 1800534 (2018). https://doi.org/10.1002/smll.201800534

[S46] S. Wu, M. Zou, Z. Li, D. Chen, H. Zhang et al., Robust and stable Cu nanowire@graphene core–shell aerogels for ultraeffective electromagnetic interference shielding. Small **14**(23), 1800634 (2018). https://doi.org/10.1002/smll.201800634
